# Tumor-associated endothelial cells display GSTP1 and RARβ2 promoter methylation in human prostate cancer

**DOI:** 10.1186/1479-5876-4-13

**Published:** 2006-03-02

**Authors:** Amelia C Grover, Michael A Tangrea, Karen G Woodson, Benjamin S Wallis, Jeffrey C Hanson, Rodrigo F Chuaqui, John W Gillespie, Heidi S Erickson, Robert F Bonner, Thomas J Pohida, Michael R Emmert-Buck, Steven K Libutti

**Affiliations:** 1 Surgery Branch, National Cancer Institute, National Institutes of Health, Bethesda, MD, USA; 2 Pathologenetics Unit, Laboratory of Pathology and Urologic Oncology Branch, Center for Cancer Research, National Cancer Institute, National Institutes of Health, Bethesda, MD USA; 3 Cancer Prevention Studies Branch, Center for Cancer Research, National Cancer Institute, National Institutes of Health, Bethesda, MD USA; 4 Laboratory of Integrative and Medical Biophysics, National Institute for Child Health and Human Development, National Institutes of Health, Bethesda, MD, USA; 5 Computational Bioscience and Engineering Laboratory, Division of Computational Bioscience, Center for Information Technology, National Institutes of Health, Bethesda, MD USA

## Abstract

**Background:**

A functional blood supply is essential for tumor growth and proliferation. However, the mechanism of blood vessel recruitment to the tumor is still poorly understood. Ideally, a thorough molecular assessment of blood vessel cells would be critical in our comprehension of this process. Yet, to date, there is little known about the molecular makeup of the endothelial cells of tumor-associated blood vessels, due in part to the difficulty of isolating a pure population of endothelial cells from the heterogeneous tissue environment.

**Methods:**

Here we describe the use of a recently developed technique, Expression Microdissection, to isolate endothelial cells from the tumor microenvironment. The methylation status of the dissected samples was evaluated for GSTP1 and RARβ2 promoters via the QMS-PCR method.

**Results:**

Comparing GSTP1 and RARβ2 promoter methylation data, we show that 100% and 88% methylation is detected, respectively, in the tumor areas, both in epithelium and endothelium. Little to no methylation is observed in non-tumor tissue areas.

**Conclusion:**

We applied an accurate microdissection technique to isolate endothelial cells from tissues, enabling DNA analysis such as promoter methylation status. The observations suggest that epigenetic alterations may play a role in determining the phenotype of tumor-associated vasculature.

## Background

Recently, the molecular study of the tumor microenvironment has taken a dramatic turn. It has now been shown in three different tissue types, breast, colon and prostate, that the cellular microenvironment surrounding the tumor epithelium actually demonstrates similar epigenetic changes to those found in the tumor cells [1-3]. Once believed to be genetically normal, evidence shows that the supporting cast of cells shares some methylation changes with the tumor cells themselves [1-3]. The meaning of these changes is not yet clear, nor is it understood how they may affect and/or support tumor progression.

While epigenetic changes have been identified in stroma, fibroblasts, and the colorectal mucosa, there has not been an evaluation of tumor-associated endothelial cells [1-3]. This is due in part to the difficulty in obtaining a pure population of endothelial cells from tissues for analysis. Endothelial cells are small, ~1 μm in diameter, and thus are not amenable to precise microdissection with current commercially available techniques. Other methods for isolating endothelial cells, such as antibody labeled magnetic beads and fluorescence activated cell sorting (FACS), require large amounts of fresh tissue and long processing times [4]. Such approaches may in themselves alter the endothelial cells, making it difficult to assess the baseline characteristics that were present in the tumor microenvironment.

To overcome this barrier, we adapted the newly described microdissection technique, Expression Microdissection (xMD), for the isolation of endothelial cells in prostate tissue [5]. Using xMD, we specifically procured endothelial cells from ethanol-fixed, paraffin-embedded whole mount prostate specimens containing both tumor and normal areas. Through the use of two antibodies for the detection of the endothelial cell population, CD31 and Factor VIII, we were able to dissect the endothelial cells away from the prostate. The goal was then to determine if endothelial cells within the tumor microenvironment demonstrated similar changes in promoter methylation to those typically seen in prostate tumor epithelial cells. Glutathione S-Transferase P1 (GSTP1) and Retinoic Acid Receptor 2 (RARβ2) were selected as markers given that their methylation has been previously shown to be more prevalent in prostate cancer cells [6-8]. Studying eight prostate cases, we demonstrate that indeed methylation of GSTP1 and RARβ2 in tumor-associated endothelium was similar to that of microdissected tumor epithelium. Moreover, the methylation rate was significantly higher in the tumor-associated endothelium compared to that of endothelial cells dissected from areas of normal prostate.

## Methods

### Tissue specimens/cell culture

Ethanol fixed paraffin-embedded prostate tumor specimens were obtained from eight different patients under Institutional Review Board (IRB) approved clinical protocols. Tissue was obtained from eight separate prostate cancer patients with clinically localized prostate cancer (organ-confined) who were treated by curative radical prostatectomy at the NIH Clinical Center and the National Naval Medical Center, Bethesda, MD.

LNCaP and PZ-HPV7 prostate cancer cell lines were obtained from American Type Culture Collection (Rockville, Maryland). LNCaP cells were maintained in RPMI 1640 medium (GIBCO-BRL, Invitrogen Corp., Carlsbad, CA) containing L-glutamine (GIBCO-BRL, Invitrogen Corp., Carlsbad, CA), 1% Antibiotics (GIBCO-BRL, Invitrogen Corp., Carlsbad, CA) and 10% heat-inactivated fetal bovine serum (FBS) (Gemini, Woodland, CA). PZ-HPV7 cells were maintained in K-SFM (Keratinocyte- Serum Free-Medium) (GIBCO-BRL, Invitrogen Corp., Carlsbad, CA) containing L-glutamine (GIBCO-BRL, Invitrogen Corp., Carlsbad, CA), 50 mg/ml BPE (GIBCO-BRL, Invitrogen Corp., Carlsbad, CA), 5 ng/ml EGF (GIBCO-BRL, Invitrogen Corp., Carlsbad, CA), and 1% Antibiotics (GIBCO-BRL, Invitrogen Corp., Carlsbad, CA). The cultures were done at 37°C, 5% CO_2 _in a humidified atmosphere.

### Expression microdissection

Eight-micron thick histological sections were mounted on charged slides. Prior to immunohistochemistry, the prostate tissue was de-waxed and rehydrated following standard techniques[9]. The DAKO Envision (+) System with diaminobenzidine (DAB) (DAKO/Cytomation, Carpinteria, CA) was used for immunohistochemistry staining following manufacturer's instructions, unless otherwise noted.

For endothelial cell staining, the combination of Mouse Anti-CD31 Ab-1 (JC/70A) 1:50 dilution (Neomarkers, Fremont, CA) and Rabbit Anti-Factor VIII 1:50 dilution (Zymed Laboratories, San Francisco, CA) was used. All antibodies were diluted in DAKO/Cytomation Antibody diluent with background reducing components (DAKO/Cytomation, Carpinteria, CA). The anti-mouse secondary antibody was incubated for 30 minutes first and then the anti-rabbit secondary antibody was incubated for an additional 30 minutes. The DAB solution was made at 3x the recommended concentration and incubated on the sections for 10 minutes. DAB enhancer (Zymed Laboratories, San Francisco, CA) was applied to the tissues for 3 minutes. The slides were not counterstained. Sections were then dehydrated through graded alcohols and xylenes and allowed to air dry.

For the epithelial dissections a 1:50 dilution of the Mouse Anti-Cytokeratin AE1/AE3 (DAKO/Cytomation, Carpinteria, CA) was used.

The laser system was an Arcturus Pix Cell II with the following parameters for the endothelium dissections: power = 70–100 mW, duration 35–50 milliseconds, repeat t = 1.2 seconds, target = 0.300V, current 25.0 mL, spot size = 7.5 μm and temperature 24°C. The parameters for the epithelium dissections were as above, except lower powers (30–50 mW) and durations (15–25 milliseconds) were used.

For each patient case, four dissected samples were generated; normal epithelium, tumor epithelium, endothelium outside the tumor area and tumor-associated endothelium.

### Bisulfite modification and QMS-PCR

The samples were digested overnight in 25 μl proteinase K buffer (10 mM Tris, pH 8.0, 1 mM EDTA, 1% Tween-20, and 1 mg/ml proteinase K). After 10 minute boiling, the DNA was directly used for the modification reactions. Gene specific hypermethylation status of GSTP1 and RARβ2 was determined using real-time (quantitative) methylation PCR (QMS-PCR) as previously described based on the Taqman Chemistry (Applied Biosystems, Foster City, CA) [7]. The primer and probe sequences have been previously published [8]. The beta-actin gene (ACTB) was used as an internal reference control.

The assays were carried out in a reaction volume of 10 μl in 96-well plate in an Applied Biosystems 7900 Sequence Detector (Perkin-Elmer, Foster City, CA). Each sample was run in triplicate and only samples with an internal reference Ct score under 42 were included in the analysis. The final reaction samples consisted of 300 nM of each primer and 100 nM probe, 1 × Taqman Universal Master Mix, No AmpErase UNG (Applied Biosystems, Foster City, CA). The PCR conditions were 95°C for 2 min, followed by 50 cycles at 95°C for 15 sec and 60°C for 1 min. Serial dilutions from 1 ng to 0.01 ng of methylated and unmethylated DNA (LNCaP prostate cancer and PZHPV7 normal prostate epithelial cell lines, respectively) was run on each plate as standards, as well as water blanks for no template controls. The assessment of percent gene methylation was computed using the standard curve method, as previously described [7]. The ratios of gene methylation relative to total amount of DNA were computed by extrapolation from standard curve of LNCaP standards (GSTP1 and RARβ2 are fully methylated in LNCaP cells). The percentages were derived using the following formula: ng gene (average value across triplicates)/ng ACTB (average value across triplicates)*100.

## Results

### Dissection of ethanol-fixed paraffin embedded whole mount prostate cancer specimens for DNA extraction

Dissections were carried out utilizing the newly developed technique, xMD [5]. Briefly, xMD employs the use of a targeting moiety, such as an antibody, to define the cell type or component to be dissected. A histological tissue section is stained via a standard immunohistochemistry protocol using diaminobenzidine (DAB) as the chromogen. The tissue is then covered with a contrast sensitive film and the area of interest on the slide, i.e. tumor or normal, is irradiated with an infrared laser procuring only the immuno-defined cells. Through this process it is possible to obtain high-quality precise dissections without the introduction of human targeting error.

**Figure 1:**
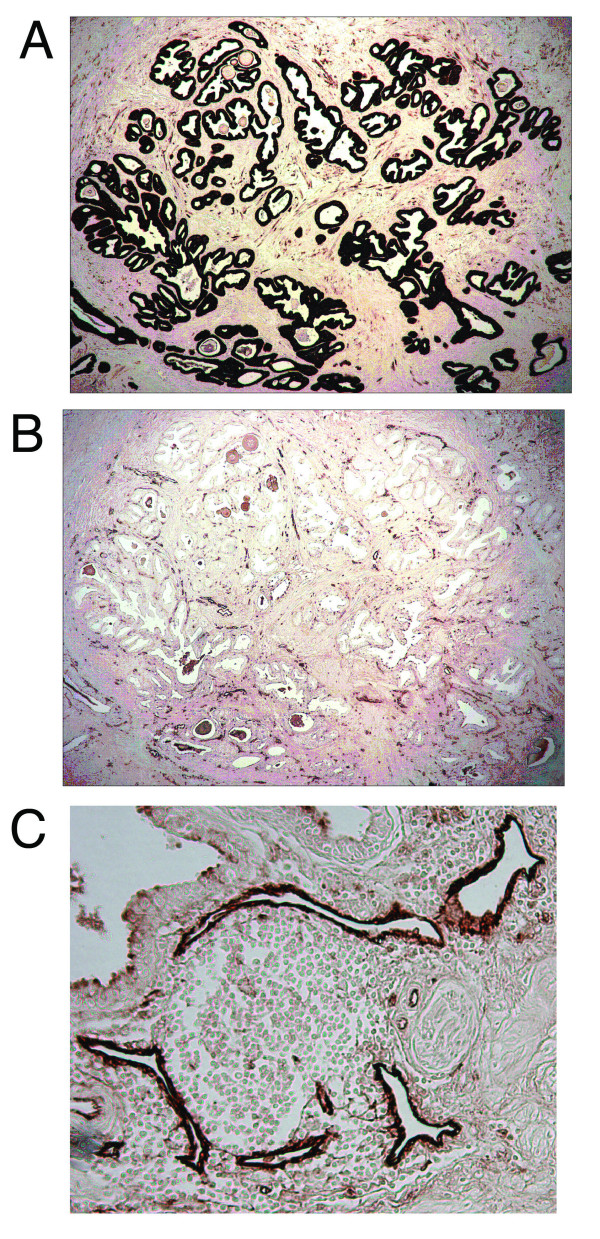
**Immunohistochemistry of the Prostate**. Representative photos of the immunohistochemistry staining of the epithelium (A) and endothelium (B) of the same area of tumor using diaminobenzidine staining. Panel C is a higher magnification of the endothelial staining to better demonstrate the dark staining of the endothelial cells.

Since endothelial cells had not been dissected previously with this method, it was necessary to optimize the immunohistochemical staining parameters for the adequate activation of the film. Previous work had demonstrated adequate immunohistochemical staining for dissection of epithelium using only single antibodies[5]. However, endothelial cells represent a much less abundant cell population comprised of only a single layer. Therefore, it was found that a combination of CD31 (1:50 dilution) and Factor VIII (1:50 dilution) antibodies was required to obtain strong enough staining for dissection while maintaining a minimal background. Figure 1 displays a representative prostate tissue stained with epithelium and endothelium specific markers after optimization. Using xMD, both the endothelium and epithelium were dissected for the neoplastic and normal areas of the prostate on the same slide for each individual patient specimen.

**Figure 2:**
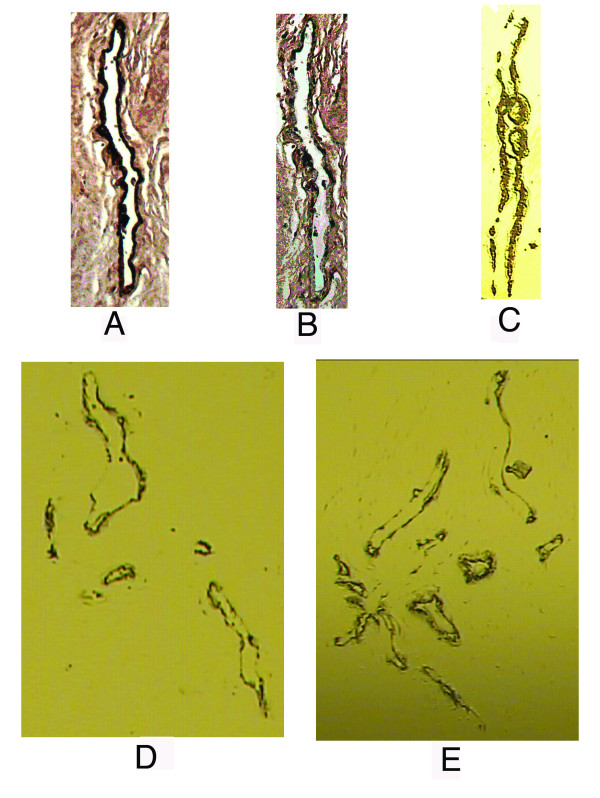
**Expression Microdissection of the Prostate Endothelium**. Representative photos of the endothelial stained slide before xMD dissection (A), after dissection (B) and the film used for dissection (C). In panel B, the darkly stained endothelial cells have been procured from the slide. Panels D and E are additional photos of endothelial cell dissections using xMD.

Figure 2 shows the tissue before dissection, after dissection and the procured dissected cells on the film. The dark staining of the endothelium is required to allow optimal dissection of the endothelial cells (Figure 2A). After dissection, the dark staining is no longer visible indicating that the endothelial cells were captured on the film (Figure 2B). The darkly stained endothelium is clearly visible on the film, maintaining its original architecture (Figure 2C). Figure 2D and 2E show further examples of xMD films with captured endothelial cells. It is thus possible to procure complete endothelial linings away from the vessels of the sections using this method of microdissection in a rapid, high-throughput manner. Furthermore, since tissue architecture is maintained, visual confirmation of adequate capture is possible.

### Increased methylation in endothelial and epithelial tissues in tumor areas

**Figure 3:**
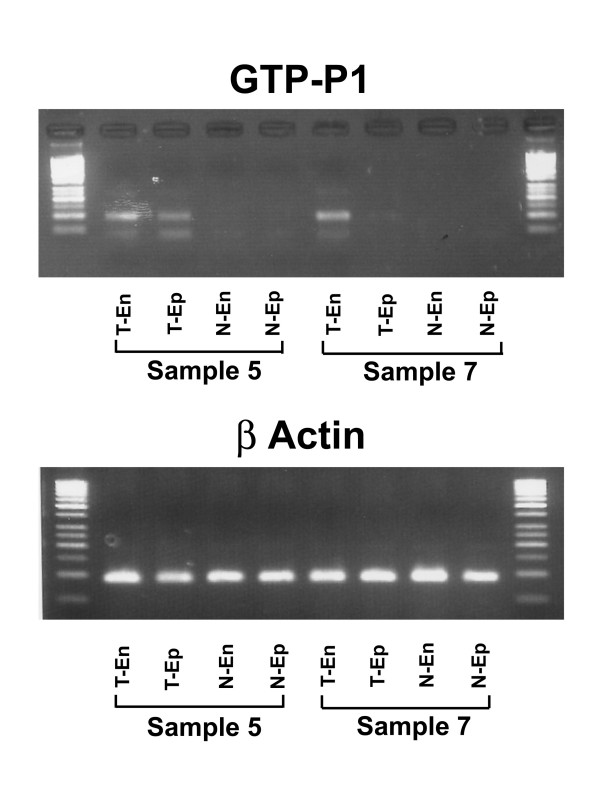
**Methylation Specific PCR**. The products from the methylation specific PCR run on agarose gels for two representative dissections are shown. In sample 5 the tumor endothelium demonstrated 75% methylation while the tumor epithelium had only 60% methylation. In sample 7 the tumor endothelium had 100% methylation while the tumor epithelium had only 10% methylation. All methylation values were normalized to β-actin. (T-En = tumor endothelium, T-EP = tumor epithelium, N-En = normal endothelium, N-Ep = normal epithelium.)

DNA was isolated from the dissected cell populations and tested for promoter methylation at GSTP1 and RARβ2 using Quantitative Methylation-Sensitive PCR (QMS-PCR) technology. Table 1 summarizes the results of the methylation status for the eight patients studied. For GSTP1, all samples demonstrated methylation in both the tumor-associated endothelium and tumor epithelium. None of the normal epithelium samples demonstrated methylation at GSTP1 and only two cases demonstrated methylation in the normal endothelium. In the tumor tissue, methylation was identified in seven out of the eight specimens for RARβ2 in both the endothelium and epithelium, while there was no methylation in the normal tissue for either cell population. To evaluate this further, the percent of methylation for the endothelium and epithelium was semi-quantified utilizing the standard curve method as previously described [7] (Table 2). While methylation was identified in both the epithelium and endothelium, the percentage of methylation varied between the two cell types. In three patients, we observed that the tumor endothelium demonstrated a higher degree of methylation when compared to the tumor epithelium. For example, the endothelium in the tumor area of patient 7 demonstrated 100% methylation at GSTP1, while the tumor epithelium only showed 10% methylation. These findings, together with the visualization of the dissected fields, support the selectivity of these dissections and the purity of the population obtained. A representative gel of methylation specific PCR products from two of the prostate cases is shown in Figure 3.

**Table 1:** Summary data of gene methylation in tumor and normal epithelium and endothelium from 8 whole mount prostate specimens.

**Histology**	**Tissue**	Gene Methylation Positive* No. (%)	
		
		**GSTP1**	**RARβ2**
Tumor (n = 8)	Endothelium	8 (100%)	7 (88%)
	Epithelium	8 (100%)	7 (88%)
Normal (n = 8)	Endothelium	2 (25%)	0 (0%)
	Epithelium	0 (0%)	0 (0%)

*Gene methylation determined by QMS-PCR and considered positive if any methylation observed.

**Table 2:** Gene Methylation in Tumor and Normal Epithelial and Endothelial Tissue from 8 Prostate Cancer Patients Determined from Whole Mount Prostate Specimens.

**Patient**	**Histology**	**Tissue Type**	**GSTP1**	**RARβ2**
1	Normal	Endothelium	0%	0%
	Normal	Epithelium	0%	0%
	Tumor	Endothelium	10%	25%
	Tumor	Epithelium	100%	100%
				
2	Normal	Endothelium	0%	0%
	Normal	Epithelium	0%	0%
	Tumor	Endothelium	100%	100%
	Tumor	Epithelium	100%	10%
				
3	Normal	Endothelium	100%	0%
	Normal	Epithelium	0%	0%
	Tumor	Endothelium	100%	100%
	Tumor	Epithelium	100%	100%
				
4	Normal	Endothelium	0%	0%
	Normal	Epithelium	0%	0%
	Tumor	Endothelium	10%	10%
	Tumor	Epithelium	100%	100%
				
5	Normal	Endothelium	0%	0%
	Normal	Epithelium	0%	0%
	Tumor	Endothelium	100%	10%
	Tumor	Epithelium	100%	25%
				
6	Normal	Endothelium	0%	0%
	Normal	Epithelium	0%	0%
	Tumor	Endothelium	100%	100%
	Tumor	Epithelium	100%	10%
				
7	Normal	Endothelium	0%	0%
	Normal	Epithelium	0%	0%
	Tumor	Endothelium	100%	100%
	Tumor	Epithelium	10%	0%
				
8	Normal	Endothelium	100%	0%
	Normal	Epithelium	0%	0%
	Tumor	Endothelium	100%	100%
	Tumor	Epithelium	100%	100%

## Discussion

Epigenetic changes have been well documented in the progression of prostate cancer [10]. In particular, GSTP1 and RARβ2 have been found to be highly methylated within the tumor epithelium of the prostate [6,11]. While there has been significant attention paid to the study of tumor cell methylation, few groups have evaluated methylation changes of the tumor-associated stromal compartment. Several cell types make up the stromal elements within the tumor microenviroment, including fibroblasts, inflammatory cells and endothelial cells.

The phenotype of tumor-associated endothelium differs dramatically from that of endothelial cells in non-tumor tissue[12]. Recent studies have also revealed gene expression changes between tumor-associated endothelial cells as compared to their counterparts in adjacent areas of normal tissue [4]. Although, epigenetic differences such as changes in methylation patterns have not been demonstrated for endothelial cells. If such differences exist, a better understanding of differential gene expression, through promotor modulation may be possible.

Using a newly developed microdissection technique, xMD, we were able to procure pure populations of endothelial cells from both the tumor microenvironment and normal areas of the prostate from the same patient specimens. The isolation of such small targets from complex human tissues has been a major obstacle when trying to analyze these cells on a molecular level. Subsequently, we identified, via bisulfite modification and QMS-PCR analysis, promoter methylation changes between tumor-associated endothelium and normal endothelium for GSTP1 and RARβ2. The observed prevalence of methylation in the tumor-associated and normal endothelial cell populations reflected the differences seen between the tumor epithelium and normal epithelium. However, there were also finer differences in methylation prevalence observed between tumor epithelium and tumor endothelium within several cases.

One issue of concern when evaluating the percent methylation was the possibility of contamination with tumor epithelial cells in the endothelial cell dissections. This is an important issue, since a false positive result may be observed if significant numbers of tumor epithelial cells were inadvertently captured during dissection. Since this is a DNA based method, it is difficult to obtain a discriminating molecular marker providing conclusive evidence of cell purity. In addition, the tissue studied was ethanol-fixed paraffin-embedded limiting our ability to perform a robust mRNA analysis. However, several factors support the integrity of the samples including; microscopic visualization of the procured endothelial cells, the lack of visual evidence of contamination by other cell populations, and the observation of varying levels of methylation between the tumor-associated endothelium and tumor epithelium within the same case, as seen in patients 2, 6, and 7. This is especially evident in cases where the percent endothelial cell methylation was higher than the percent epithelial cell methylation. Additionally, the specificity of the xMD technique was demonstrated previously by the ability to dissect specific small targets, such as nuclei and basal cells in prostate tissue [5].

It is also interesting to note that two patient samples displayed 100% methylation for GSTP1 in the isolated normal endothelial cell population. Considering this finding, we surveyed the tissue specimens for histological features that might be unique to those cases, including the amount and type of inflammatory infiltrate in the stroma. None were observed. A mild, focal chronic inflammatory infiltrate was seen in all cases. Hence, the significance of this finding remains unclear.

While others have evaluated the effect of promoter methylation on gene expression in endothelial cells, this is the first report of differential endothelial promoter methylation in the tumor microenvironment. Growing evidence has shown a possible field effect of the tumor cells on their surrounding microenvironment [1-3]. The fact that the tumor-associated vessels demonstrate differential methylation when compared to normal vessels suggests that methylation may play some role in establishing the unique phenotype of the tumor vasculature. Such an observation, if supported by further studies, could allow for the identification of new targets for the development of therapies directed to the tumor vasculature.

## Competing interests

MAT, RFC, RFB and MREB are inventors listed on the expression microdissection patent application. All other authors declare that they have no competing interests.

## Authors' contributions

ACG carried out the microdissections, participated in the methylation studies and drafted the manuscript, MAT participated in the microdissection process and helped draft the manuscript, KGW carried out the methylation studies, BSW participated in the microdissection process, JCH participated in the methylation studies, RFC evaluated tissue specimens, JWG evaluated tissue specimens, HSE participated in the immunohistochemistry analysis, RFB participated in the microdissection process, TJP participated in the microdissection process, MREB participated in the design of the study, and SKL conceived of the study, and participated in its design and coordination and helped to draft the manuscript. All authors read and approved the final manuscript.
